# Molecular Characterization and Functions of Fatty Acid and Retinoid Binding Protein Gene (*Ab-far-1*) in *Aphelenchoides besseyi*

**DOI:** 10.1371/journal.pone.0066011

**Published:** 2013-06-05

**Authors:** Xi Cheng, Yu Xiang, Hui Xie, Chun-Ling Xu, Teng-Fei Xie, Chao Zhang, Yu Li

**Affiliations:** Laboratory of Plant Nematology and Research Center of Nematodes of Plant Quarantine, College of Natural Resources and Environment, South China Agricultural University, Guangzhou, People's Republic of China; Russian Academy of Sciences, Institute for Biological Instrumentation, Russian Federation

## Abstract

Rice white tip nematode, *Aphelenchoides besseyi*, is a kind of plant parasitic nematodes that cause serious losses in rice and many other crops. Fatty acid and retinoid binding protein (FAR) is a specific protein in nematodes and is related to development, reproduction, infection to the host, and disruption of plant defense reactions, so the inhibition of FAR function is the potential approach to control *A. besseyi*. The full-length of *Ab-far-1* cDNA is 805 bp, including 546 bp of ORF that encodes 181 amino acids. Software analysis revealed that the *Ab*-FAR-1 was rich in α-helix structure, contained a predicted consensus casein kinase II phosphorylation site and a hydrophobic secretory signal peptide, but did not have glycosylation sites. The *Ab*-FAR-1 had 52% homology to *Gp*-FAR-1 protein. The *Ab*-FAR-1 and *Gp*-FAR-1 were grouped in the same branch according to the phylogenetic tree. Fluorescence-based ligand binding analysis confirmed that the recombinant *Ab*-FAR-1 (r*Ab*-FAR-1) bound with the fluorescent analogues 11-((5-dimethylaminonaphthalene-1-sulfonyl) amino) undecannoic acid (DAUDA), cis-parinaric acid and retinol, but the oleic acid would compete with the binding site. Quantitative PCR (qPCR) was used to assess the expression level of *Ab-far-1* at different development stages of *A. besseyi*, the highest expression was found in the females, followed by eggs, juveniles and males. Using *in situ* hybridization technique, *Ab-far-1* mRNA was present in the hypodermis of juveniles and adults, the ovaries of females and the testes of males. When *A. besseyi* was treated with *Ab-far-1* dsRNA for 48 h, the silencing efficiency of *Ab-far-1* was the best and the number of nematodes on the carrot was the least. Thus FAR plays important roles in the development and reproduction of nematodes. This study is useful and helpful to figure out a new way to control the plant parasitic nematodes.

## Introduction

Rice white tip nematode, *Aphelenchoides besseyi*, is a kind of foliar nematodes that feed ecto- or endoparasitically above-ground parts of plant, it can parasite in more than 200 kinds of plants in 35 genera. Rice (*Oryza sativa*) and strawberry (*Fragaria ananasa*) are the most common hosts [1], [2]. *A. besseyi* is widely distributed and occurs in most rice growing areas of the world, and rice yield is reduced by 10%–71% in the occurred paddies [3]. At present, chemical seed treatment and soil application are the main approaches to control *A. besseyi*. But there are some risks of damaging the germination after treatment. On the other hand, chemical nematicide has not been recommended due to its high toxicity.

With the development of molecular biology, the genetic engineering technology has been applied in the genome research of plant parasitic nematodes. The molecular methods are widely used to study the effective and safe way to prevent plant parasitic nematodes. Therefore, the studies on the genes that are related to the life activity and infection mechanism of plant parasitic nematodes are very important.

In the processes of development and infection to the host, plant parasitic nematodes require fatty acids and retinol for lipid biosynthesis and assembly of macromolecular structures, but they are unable to synthesize fatty acids and retinol by themselves. To sustain their life activities, plant parasitic nematodes have to obtain these metabolites from their hosts and the environment through the lipid binding proteins (LBPs) [4], [5]. Nematodes have been found to produce a series of unusual proteins that exhibit high affinity binding to lipid, and these proteins can be divided into two different classes according to their molecular weight and structure features: polyprotein allergens/antigens (NPAs) and fatty acid and retinoid binding proteins (FARs) [6], [7]. FAR proteins which are rich in helix structure have different structure from proteins of similar biochemical function from other organism groups. FARs promote the absorption, transportation and specific localization of fatty acid and retinoid [4], [8]. Retinol plays important roles in gene activation, cell signaling, and tissue differentiation and repairation [5], [7], [8]. Thus the secreted FARs can help nematodes not only to obtain the lipid nutrition from the host, but also to infect the host and inhibit the host defense mechanism. The first identified FAR protein was the *Ov*-FAR-1 (formerly known as *Ov*20) from *Onchocerca volvulus* [9]. *Ov*-FAR-1 can promote the infection by reducing the defense of host, and cause worse damage to the host [9], [10]. Eight FARs have been identified in the model nematode *Caenorhabditis elegans*, named *Ce*-*far-*1 to 8 [7]. FARs have also been found in animal parasitic nematodes, including *Ancylostoma caninum*, *Brugia malayi*, *Brugia pahangi*, *Ancylostoma ceylanicum*, *Acanthocheilonema viteae*, *Ascaris suum*, *Heligmosomoides polygyrus*, *Onchocerca gutturosa*, *Onchocerca dukei*, *Onchocerca ochengi*, *Loa loa*, *Wuchereria bancrofti*, and *Litomosoides sigmodontis* [5], [8], [11], [12], [13], [14]. In plant parasitic nematodes, however, only one FAR from *Globodera pallida* (*Gp*-FAR-1) has been identified, which is present in the hypodermis of second-stage juvenile and is related to the defense system of plant host at the initial infection stage [15].

FAR plays a critical role in the development and infection processes of plant parasitic nematodes [15]. As an effective target, FAR has been attracted much attention to control plant parasitic nematodes. According to the expressed sequence tag (EST) of FAR gene from cDNA library of *A. besseyi*, we amplified the full-length cDNA of FAR gene from *A. besseyi* (*Ab-far-1*) and analyzed the structure and feature of *Ab-far-1* gene with the bioinformatic method. We used fluorescence-based binding assays to investigate the binding activity of bacterial recombinant *Ab*-FAR-1 to fatty acid and retinol. We localized the *Ab-far-1* mRNA at different development stages by *in situ* hybridization and detected the expression level of *Ab-far-1* by quantitative PCR (qPCR). In addition, the function of *Ab-far-1* gene was investigated by RNA interference (RNAi) approach. This is the first study to identify and analyze the *Ab-far-1* gene, and to investigate its function using RNAi technique.

## Materials and Methods

### Ethics statement

We collected the nematodes in areas where rice white tip nematodes occurred and no specific permit was required. The field for nematodes collection was neither privately owned nor protected, and did not involve endangered or protected species.

### Nematodes

*A. besseyi* used in this study was collected from the leaves of infected *O. sativa* in Nanjing City, Jiangsu Province, China where *A. besseyi* occurred and identified by laboratory of plant nematology, South China Agricultural University. *A. besseyi* was preserved and cultured on excised carrot (*Daucus carota*) disks in Petri dishes at 25°C in dark incubator [3], [16].

### Nematode extraction

The carrot callus inoculated with *A. besseyi* for 30 days was mashed with a blender. The mashed solution was filtered through the combine sieves with aperture size of 0.147 mm and 0.026 mm. Nematodes were collected from 0.026 mm aperture sieve in a beaker and Petri dishes.

### Cloning of full-length FAR gene from *A. besseyi*

Total RNA of 20,000 mixed stages nematodes was extracted with TRIzol reagent (Invitrogen, Carlbard, CA, USA), then treated with RQ1 RNase-Free DNase (Promega, Madison, WI, USA) at 37°C for 15 min. The cDNA sequences were amplified by a SMART RACE cDNA Amplification kit (Clontech, Japan). According to the EST sequences of *A. besseyi* FAR gene that was screened from the cDNA library of *A. besseyi* in our lab (unpublished, Fig. S1), 5' RACE primers (FAR-R1 and FAR-R2) and 3' RACE primers (FAR-F1and FAR-F2) (Table 1) were designed to amplify the cDNA sequence. The amplification products were purified and ligated into the pMD 18-T vector (Takara, Japan), and then transformed into *Escherichia coli* JM109 competent cells. The positive clones were sent to BGI Company for sequencing. Based on the sequencing results of the 5' and 3' RACE products, the specific primers of QCFF and QCFR (Table 1) were designed and used to amplify the full-length cDNA of *A. besseyi* FAR gene.

**Table 1:** Primers used in this study.

Primer	Sequence	Source
FAR-F1	5′-GAGACTTTCCCGCAAATCACC-3′	
FAR-F2	5′- ATCGACACCTACAAGAAATTGGCTG -3′	
FAR-R1	5′- GCAGATGATTGTGACCGTTTAGTTC -3′	
FAR-R2	5′- GATTGAAGCAGATGATTGTGACCGT -3′	
QCFF	5′- CGTTGTTTCAGTAACTCACATCTTT -3′	
QCFR	5′- GAACATACGAATACAACTAAAAGGA -3′	
FARf-BamHI	5′-GAGAGAGGATCCAGCCTTCGTACCTTCGTTGT-3′	
FARr-XhoI	5′-GAGAGACTCGAG GCAGATGATTGTGACCGTTTA-3′	
18sF	5′- CTCGTGGTGGCTGGTATGCTG -3′	
18sR	5′- GTTTCCCGTGTTGAGTCAAATTAAG -3′	
qPCR-F	5′- TCGCTCTTCTGTCTTGCCATG -3′	
qPCR-R	5′- GATGGATTTGTCTTCATCGGTA -3′	
FAR-IN-T7S1	5′-TAATACGACTCACTATAGGGGCCTTCGTACCTTCGTTGTCTTG -3′	
FAR-IN-A1	5′- GTTCTGGCACTTGTTGAATGCTC -3′	
FAR-IN-T7A1	5′-TAATACGACTCACTATAGGGGTTCTGGCACTTGTTGAATGCTC-3′	
FAR-IN-S1	5′- GCCTTCGTACCTTCGTTGTCTTG -3′	
FARRiF	5′- TACGCCGAACTTACCGATGAA -3′	
FARRiT7R	5′-TAATACGACTCACTATAGGGCAAGTCTGGCTTCTCTTCACCC -3′	
FARRiT7F	5′-TAATACGACTCACTATAGGGTACGCCGAACTTACCGATGAA -3′	
FARRiR	5′- CAAGTCTGGCTTCTCTTCACCC -3′	
G-T7S	5′-GGATCCTAATACGACTCACTATAGGGCACAAGTTCAGCGTGTCCGGCG-3 -3′	Zhang et al., 2012
G-A	5′- CGATGCGGTTCACCAGGGTGTCG -3′	Zhang et al., 2012
G-T7A	5′-GGATCCTAATACGACTCACTATAGGG CGATGCGGTTCACCAGGGTGTCG -3′	Zhang et al., 2012
G-S	5′- CACAAGTTCAGCGTGTCCGGCG -3′	Zhang et al., 2012

### Sequence analysis, alignment and phylogenetic studies

Sequence homology comparisons were conducted using BLASTX and BLASTN (http://blast.ncbi.nlm.nih.gov/Blast.cgi). The protein transmembrane regions, theoretical isoelectric point, molecular weight, hydrophobicity and glycosylation sites were predicted by the Protein Machine software available at the Expasy site (http://www.expasy.ch/tools/). Predictions of a signal peptide for secretion and the cleavage site were performed at http://www.cbs.dtu.dk/services/SignalP/. The prediction of protein localization site was performed at http://psort.hgc.jp/form2.html. A theoretical 3D structure of *Ab*-FAR-1 was constructed with the automated mode of SWISS-MODEL. The amino acid sequences of FAR protein from *A. besseyi* and other six FAR proteins from four species of nematodes [17] were aligned using DNAMAN software (Lynnon Biosoft, Canada). Based on the amino acid sequences of *Ab*-FAR-1 and other 25 FAR proteins from 16 species of nematodes, a phylogenetic tree was constructed by the neighbor-joining method [18] with MEGA (Molecular Evolutionary Genetics Analysis, USA).

### Expression and purification of recombinant *Ab*-FAR-1

To obtain purified *Ab*-FAR-1 protein,the full-length *Ab-far-1* was amplified from the plasmid with primers FARf-BamHI and FARr-XhoI (Table 1), then cloned into prokaryotic expression vector pET-32a (Novagen, Madison, WI, USA) after digestion with BamHI and XhoI. The plasmid was introduced into *E. coli* DH5α for sequence confirmation. Recombinant plasmid DNA was introduced into *E. coli* BL21(DE3)for expression. Expression of the recombinant protein was examined by sodium dodecyl sulfate polyacrylamide gel electrophoresis (SDS-PAGE) and Coomassie brilliant blue staining after treatment with 1 mM isopropyl β-D-thiogalactopyranoside (IPTG). The recombinant fusion *Ab*-FAR-1 protein with His-tag at the N-terminus was purified by affinity chromatography using Ni Sepharose High Performanc (GE Healthcare, Sweden) according to the manufacturer's instructions. The purity of purified recombinant protein was confirmed by SDS-PAGE.

### Ligand-binding experiments

The binding activity of purified *Ab*-FAR-1 protein to fatty acid was measured using the fluorescent analogue 11-((5-dimethylaminonaphthalene-1-sulfonyl) amino) undecannoic acid (DAUDA) (Sigma, USA), the naturally fluorescent cis-parinaric acid (Molecular Probes) (Cayman, USA), retinol (Sigma, USA) and oleic acid (Sigma, USA). Fluorescence measurements were performed at 20°C with FluoroMax 4 (HORIBA Jobin Yvon, France) in a total volume of 3 ml per well as described previously [15], [19]. The excitation wavelengths used for DAUDA, retinol and cis-parinaric acid were 345, 350 and 319 nm, respectively. DAUDA, cis-parinaric acid and oleic acid were prepared at 10 mM stock solution in ethanol. DAUDA and oleic acid were used at 1∶1000 dilutions in PBS; while cis-parinaric acid was diluted at 1∶2000 in PBS. Free retinol was freshly prepared at 10 mM in ethanol and further diluted at 1∶1000 in ethanol and added directly to the protein solutions.

Dissociation constants were estimated in fluorescence titration experiments as described previously [5], [15], [19]. The dissociation constant (Kd) for r*Ab*-FAR-1 binding to DAUDA and cis-parinaric acid were estimated by adding increasing concentrations of r*Ab*-FAR-1 to 10 µM DAUDA in PBS and 10 µM cis-parinaric acid in PBS (total volume of 3 ml), respectively. To determine the Kd for r*Ab*-FAR-1 binding to retinol, increasing concentrations of fluorescent ligand were added to 10 µM *Ab*-FAR-1 solution in Tris-HCl buffer. Fluorescence data were corrected for dilution and fitted by standard nonlinear regression techniques (using Microcal ORIGIN software) to a single noncompetitive binding model to give estimation of the dissociation constant (Kd) and maximal fluorescence intensity (Fmax).

### Expression of *Ab-far-1* mRNA at different development stages of *A. besseyi*

qPCR was used to assess the expression levels of *Ab-far-1* at different development stages of *A. besseyi*: females, males, juveniles and eggs. RNA samples were prepared from 100 nematodes at each developmental stage using the MicroElute total RNA kit (OMEGA) according to the manufacturer's instructions. Total RNA was treated with RQ1 RNase-Free DNase (Promega) as described above. The RNA was quantified by a Nano-drop spectrophotometer and stored at -80°C for further analysis. All RNAs used for qPCR were prepared in triplicate.

cDNA was synthesized by a ReverTra Ace qPCR RT kit (TOYOBO) with random primers according to the manufacturer's instructions. The specific primers qPCR-F and qPCR-R (Table 1) for *Ab-far-1* were designed to detect *Ab-far-1* expression level. The 140 bp of 18S rRNA (AY508035) was amplified as a reference gene using the primers 18sF and 18sR (Table 1). The qPCR was performed on CFX-96 (Bio-Rad) qPCR machine using SYBR Green Real-time PCR Master Mix-plus kit (TOYOBO) according to the manufacture's protocol. All assays were performed in triplicate. Initial data analysis was carried out to create Ct values using Bio-Rad CFX-96 manager software and to determine the relative expression level by the melt curves. 18S rRNA was used as a positive control in all experiments. All experiments were performed in triplicate.

### Localisation of *Ab-far-1* mRNA by using *in situ* hybridization

*In situ* hybridization was performed as described by De Boer et al. [20] with some modifications. 10,000 mixed stages nematodes, including females, males and juveniles separated from carrot callus were concentrated to a 30–50 µl pellet and fixed in 3% paraformaldehyde at 4°C for 16 h. The specific sense (FAR-IN-T7S1, FAR-IN-A1) and antisense (FAR-IN-T7A1, FAR-IN-S1) (Table 1) primers were designed to amplify DIG-labelled sense and antisense RNA probes (Roche, Germany) from full-length cDNA of *Ab*-*far*-1 gene. DIG-labelled sense or antisense RNA probe was added to the hybridization solution containing the nematode sections, and then rotated at 47°C for 12 h. After hybridization, the nematodes were examined and photographs were taken with differential interference microscopy.

### Synthesis of *Ab-far-1* dsRNA

The fragment of *Ab-far-1* ORF was cloned into the vector pMD18-T (Takara, Japan), and confirmed by sequencing. Two primer pairs of FARRiF/FARRiT7R and FARRiT7F/FARRiR (Table 1) were designed to amplify the sense and antisense single-stranded RNA (ssRNA) products. The sense and antisense ssRNAs were transcribed from *Ab-far-1* plasmid using the ScriptMAX Thermo T7 Transcription KIT (TOYOBO, Japan) according to the manufacturer's instructions, and mixed together in equal proportions and heated at 94°C for 10 min, then cooled to room temperature to allow the annealing of complementary strands. The dsRNA was purified with 1/10 amount of KAc (3 mol/l) and 2-fold amount of 95% ethanol overnight, then washed with 75% ethanol for 3 times and dissolved in deionized water. The quantity of dsRNA was measured by a Nano-drop spectrophotometer and analyzed by 1.2% agarose gel electrophoresis, and finally stored at −80°C for later use. Non-endogenous control dsRNA (125 bp) (green fouorescent protein gene, *gfp*) was generated with specific primers (G-T7S, G-A, G-T7A, and G-S) (Table 1) [21].

### Knockdown of *Ab-far-1* using dsRNA

500 mixed stage nematodes separated from carrot callus were collected in a DEPC-treated Eppendorf tube and washed twice with DEPC water, and then soaked in 50 µl *Ab-far-1* dsRNA solution (2 µg/μl) and lightly shook in a rotary incubation (200 rpm) for 12 h, 24 h, 36 h and 48 h in the dark at 25°C. Non-endogenous *gfp* dsRNA solution 50 µl (2 µg/μl) was used as a control. After soaking in dsRNA, the nematodes were washed three times with DEPC water and the total RNA was then extracted by the MicroElute total RNA kit (OMEGA) as described above. qPCR was used to analyze the transcript suppression of *Ab-far-1* in *A. besseyi* after dsRNA treatment. All assays were performed in triplicate.

To test the reproductive capacity of *Ab-far-1* silenced nematodes, thirty female nematodes separated from carrot callus were treated with dsRNA for 12 h, 24 h, 36 h and 48 h respectively. Then all nematodes were inoculated onto carrot callus and maintained in a dark incubator at 25°C for 35 days. Isolating and calculating the number of total nematodes in the carrot callus at last. Each treatment was carried out in 5 duplicates. All data in this study were subjected to analysis of variance (ANOVA) and multiple comparisons of means were conducted by Duncan's Multiple Range Test at *p* = 0.01 using SAS (Release 8.01).

## Results

### Cloning of full-length FAR gene from *A. besseyi*

According to the EST sequences (486 bp) of FAR gene from cDNA library of *A. besseyi*, the specific primers for FAR gene were designed to amplify the 805 bp full-length cDNA sequence and cloned into pMD 18-T vector. The plasmid was named *Ab-far-1*, which included a 546 bp of open reading frame (ORF) for encoding a deduced 181 amino acids (GenBank accession number JQ686690**)** (Fig. S2). The ORF began with an ATG initiation codon at nucleotide 93 and terminated with TAA at nucleotide 638 (Fig. S2).

### Sequence analysis of the *Ab*-FAR-1 protein

The *Ab*-FAR-1 protein encoded 181 amino acids with theoretical molecular mass of 20.5217 kDa, the molecular formula was C_923_H_1514_N_238_O_280_S_3_ with a theoretical isoelectric point of 6.66. The location site of *Ab*-FAR-1 protein was predicted in extracellular compartment (including cell wall). *Ab*-FAR-1 sequences had the highest similarity with the *Gp*-FAR-1 protein from *G. pallid*a (GenBank accession number CAA70477.2, 52% identity and 73% similarity, E-value 1e-51) and the FAR from *O. ochengi* (GenBank accession number ACB70198.1, 51% identity and 73% similarity, E-value 1e-54). *Ab*-FAR-1 also showed higher similarities with the *Ov*-FAR-1 from *O. volvulus* (GenBank accession number Q25619, 50% identity and 73% similarity, E-value 8e-56) and *Og*-FAR-1 from *O. gutturosa* (GenBank accession number Q8WT59, 50% identity and 73% similarity, E-value 1e-55).

Like other six FARs from four species of nematodes [17], *Ab*-FAR-1 protein contained a hydrophobic secretory signal peptide and was rich in α-helix but no extended β structure (Fig. S3). Moreover, the *Ab*-FAR-1 also contained a conserved casein kinase II phosphorylation site, but did not have the glycosylation sites like *Ce*-FAR-1 and *Ce*-FAR-2.

**Figure 1:**
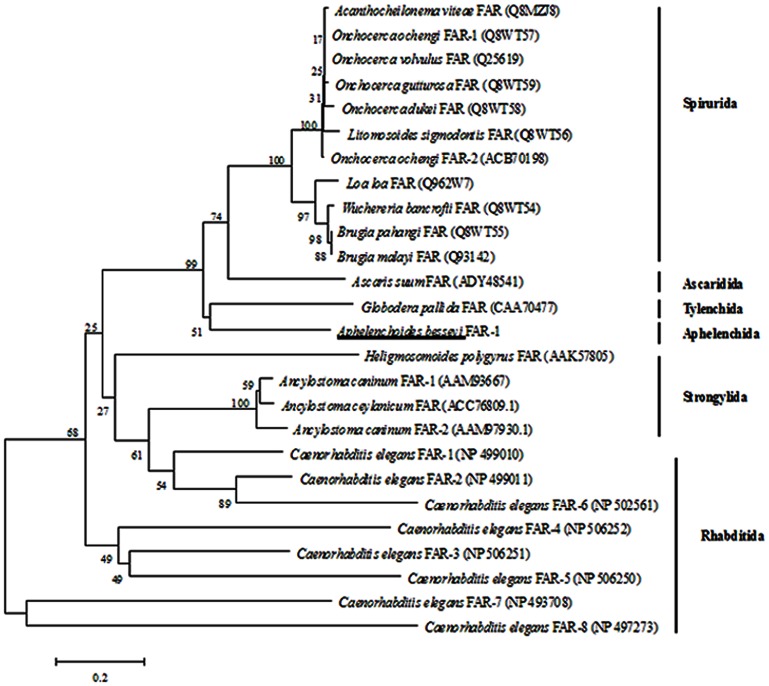
Phylogenetic relationships among the amino acid sequences of fatty acid and retinoid binding proteins in nematodes. Phylogram was constructed according to amino acid sequences depicting the evolutionary relationships among fatty acid and retinoid binding proteins of 26 different nematode species, *Aphelenchoides besseyi* FAR-1 highlighted by the underline. Accession number of the sequences was in brackets.

In the phylogenetic tree (Fig. 1) constructed by the *Ab*-FAR-1 amino acid sequences and other 25 FAR proteins from 16 species of nematodes, *Ab*-FAR-1 of Aphelenchida nematode *A. besseyi* and *Gp*-FAR-1 of Tylenchida nematode *G. pallida* were present in the same branch of the phylogenetic tree, which suggests that they have a closer genetic relationship. In addition, all these 26 FAR proteins from 17 species of nematodes were divided into 6 groups: Spirurida, Ascaridida, Tylenchida, Aphelenchida, Strongylida, and Rhabditida.

### Ligand binding

**Figure 2:**
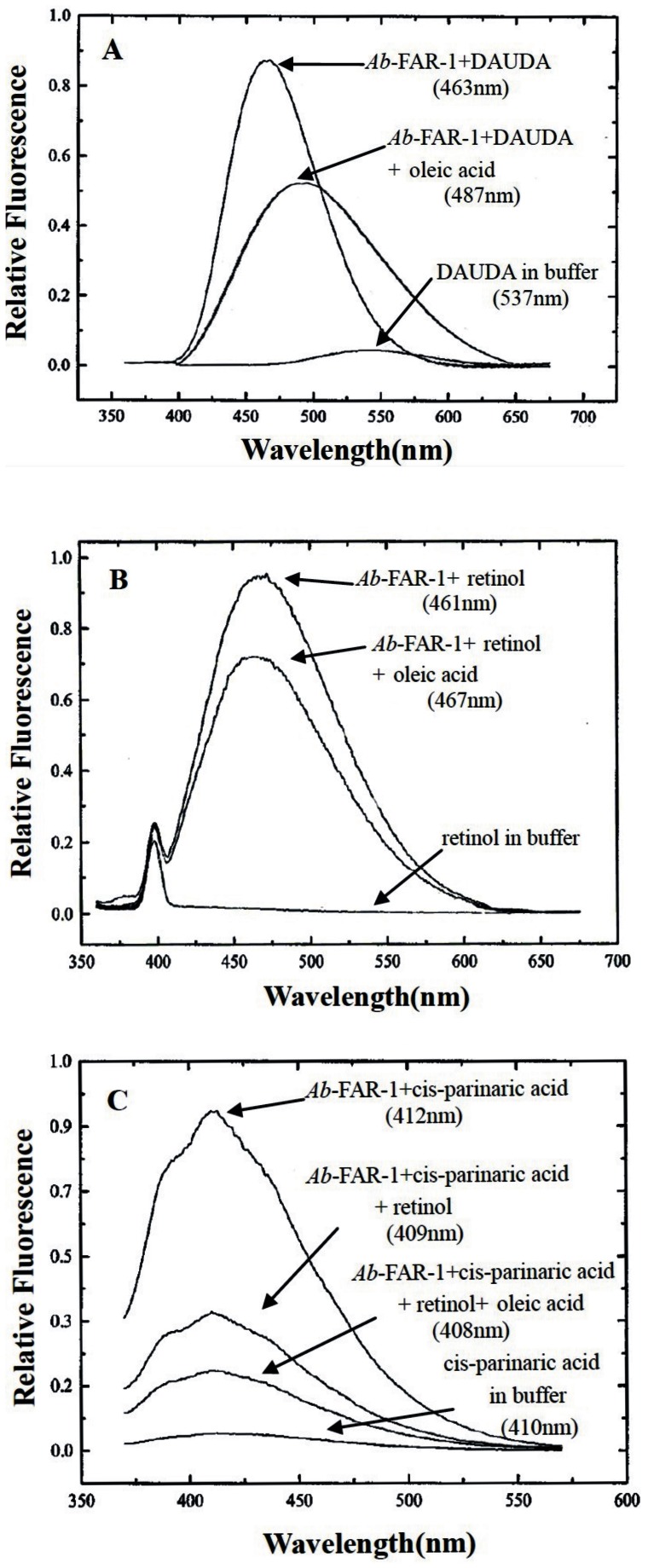
Ligand binding by r*Ab*-FAR-1. **(A)** *Ab*-FAR-1. The reverse change of DAUDA emission was observed after the addition of oleic acid to the r*Ab*-FAR-1+DAUDA complex. The wavelengths of peak emission by DAUDA were different. **(B)** Fluorescence emission spectra (excitation at 350 nm) of retinol in ethanol alone or after the addition of r*Ab*-FAR-1. The competitive effect of oleic acid was also found. **(C)** Fluorescence emission spectra (excitation at 319 nm) of cis-parinaric acid alone or after the addition of r*Ab*-FAR-1.The effects of the subsequent addition of retinol to the r*Ab*-FAR-1+cis-parinaric acid and addition of oleic acid solution to the r*Ab*-FAR-1+cis-parinaric acid+ retinol were examined.

SDS-PAGE showed that the recombinant *Ab*-FAR-1 was well purified, with only one band of approximately 19-20 KDa, which was consistent with theoretical molecular mass (20 KDa) of *Ab*-FAR-1 (Fig. S4). The purified r*Ab*-FAR-1 was found to bind the dansylated fatty acid DAUDA because a significant wavelength shift of peak fluorescence emission was observed (Fig. 2A). In buffer alone, the peak emission of DAUDA occurred at 537 nm, but moved to 463 nm upon addition of r*Ab*-FAR-1 (Fig. 2A). The wavelength shift indicates that r*Ab*-FAR-1 has a highly apolar binding site, and the degree of shift is unusually large for lipid transporter proteins but typical for FAR protein. These characteristics have been demonstrated by previous studies [13], [15], [17], [22], [23], [24], [25], [26]. After the addition of oleic acid to DAUDA, protein complex produced a pronounced drop in the fluorescence intensity (487 nm) (Fig. 2A), indicating the oleic acid can displace DAUDA from the binding site. The retinol binding activity of purified *Ab*-FAR-1 test showed that the fluorescence emission of retinol was minimal in buffer alone, but was substantially increased to 461 nm when the retinol was added to the r*Ab*-FAR-1 solution (Fig.2B). After the addition of oleic acid to retinol, the protein complex produced an obvious drop in the fluorescence intensity (467 nm) (Fig.2B). r*Ab*-FAR-1 was also found to bind the intrinsically fluorescent cis-parinaric acid. In control buffer, the emission peak of cis*-*parinaric acid appeared at 410 nm, but moved to 412 nm upon addition of r*Ab*-FAR-1 (Fig.2C). The effects of subsequent addition of retinol to the r*Ab*-FAR-1+cis-parinaric acid and addition of oleic acid solution to the r*Ab*-FAR-1+cis-parinaric acid+ retinol were different (Fig.2C). These results indicate that the retinol and fatty acid binding sites are congruent, overlapping, or interactive, and the addition of oleic acid can competitively displace cis*-*parinaric acid.

**Figure 3:**
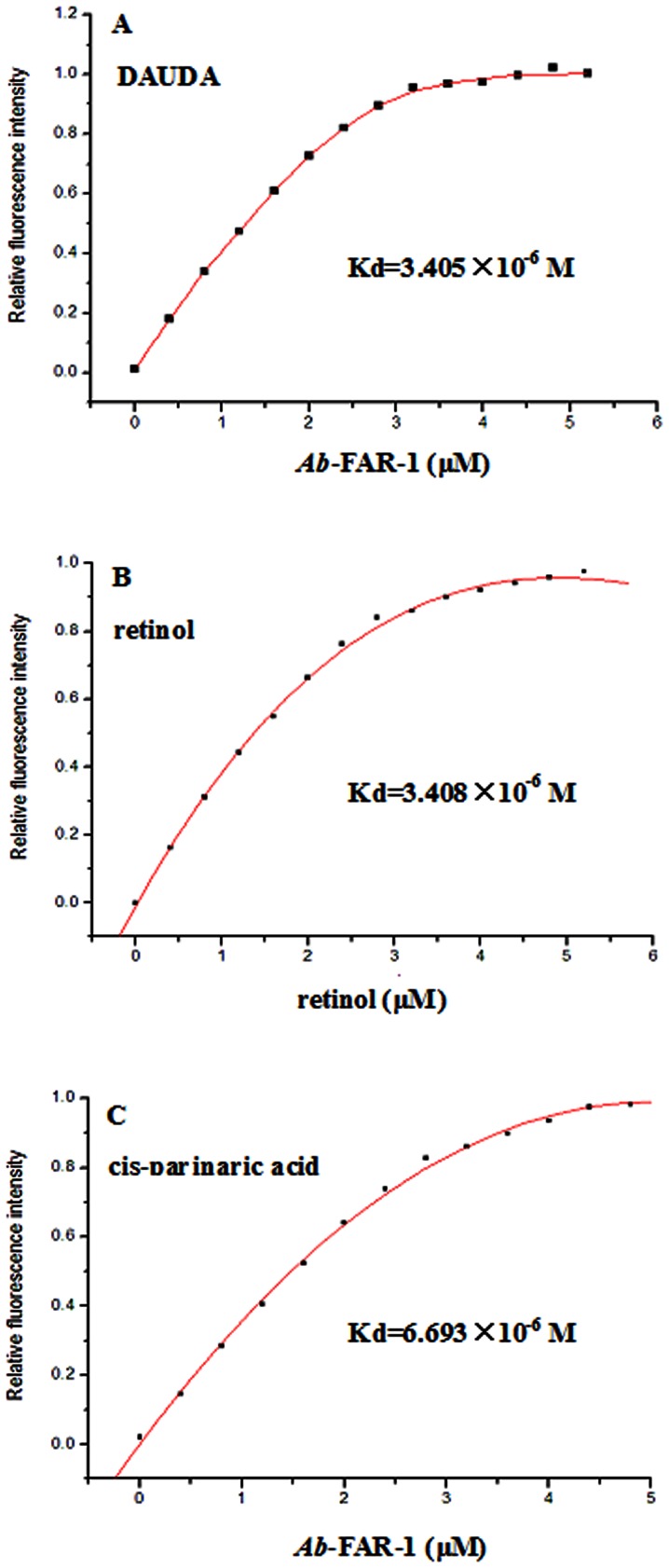
Fluorescent titration lipid binding analysis of r*Ab*-FAR-1. **(A)** Change in relative fluorescence intensity (excitation at 345 nm) of DAUDA (10 µM) in the presence of increasing concentrations of r*Ab*-FAR-1. The best fit curve was used to determine the dissociation constant (Kd) for the DAUDA: r*Ab*-FAR-1 interaction. **(B)** Change in relative fluorescence intensity (excitation at 350 nm) of 10 µM r*Ab*-FAR-1 in the presence of increasing concentrations of retinol. The curve was used to derive the equilibrium dissociation constant (Kd) for the retinol: r*Ab*-FAR-1 interaction. **(C)** Change in relative fluorescence intensity (excitation at 319 nm) of cis-parinaric acid (10 µM) in the presence of increasing concentrations of r*Ab*-FAR-1. The best fit curve was used to determine the dissociation constant (Kd) for the cis-parinaric acid: r*Ab*-FAR-1 interaction.

Fluorescence titration experiments were performed to determine the binding affinity of DAUDA, retinol and cis-parinaric acid. Figure 3 showed typical saturation binding curves for each of the three fluorescent ligands. The titration curve (corrected for the background fluorescence of DAUDA) in Figure 3A predicted a Kd of 3.405×10^−6^ M for the *Ab*-FAR-1: DAUDA interaction. We also demonstrated binding of *Ab*-FAR-1 to retinol (Kd of 3.408×10^−6^ M) (Fig. 3B) and cis-parinaric acid (Kd of 6.693×10^−6^ M) (Fig. 3C). These values were within the general range of dissociation constants reported for other FAR proteins [5], [7], [8], [15], [19]. The results indicate that *Ab*-FAR-1 is a functional protein and has binding activities to fatty acid and retinol in vitro.

### Expression and localization of *Ab-far-1* mRNA

**Figure 4:**
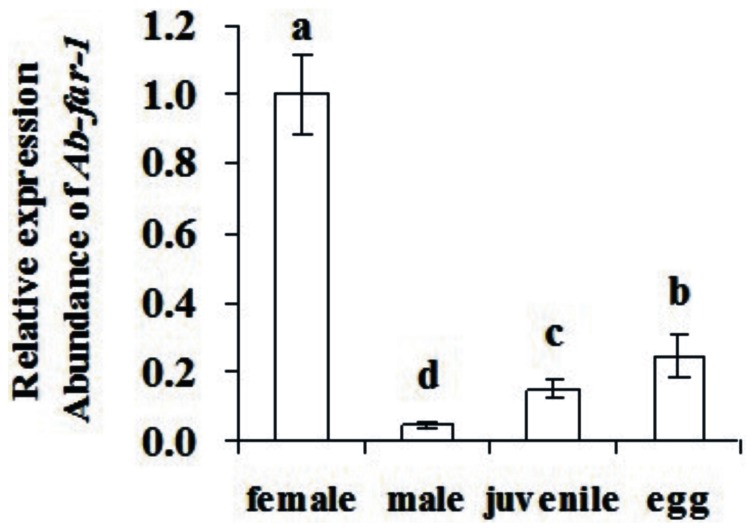
Expression of the *Ab-far-1* in 100 eggs, females, juveniles and males, respectively of *Aphelenchoides besseyi*. Bars indicate standard errors of mean data (n = 3) and different letters indicate significant differences (*p*<0.01) between treatments.

**Figure 5:**
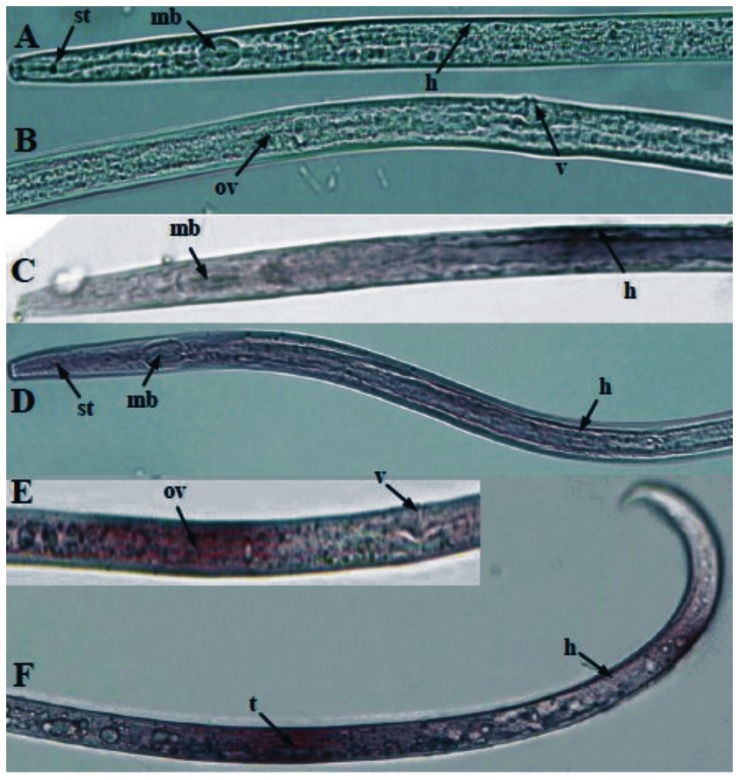
Tissue localisation of *Ab-far*-1 mRNA in juveniles, females and males of *Aphelenchoides besseyi* using *in situ* hybridization. (**A** and **B**) No signal in *A. besseyi* section was hybridized with sense *Ab-far*-1 DIG- Labeled RNA probe. (**C**) *Ab-far*-1 was present in the hypodermis of juveniles. (**D**) *Ab-far*-1 was present in the hypodermis of females. (**E**) *Ab-far*-1 was present in the ovaries of females. (**F**) *Ab-far*-1 was present in the hypodermis and testes of males. st: stylet, mb: medium bulb, h: hypodermis, ov: ovaries, v: vulva, t: testis.

The qPCR results showed that *Ab-far-1* mRNA transcript was present in all developmental stages, the highest transcript levels noted in females, while its expression in eggs, juveniles and males accounted for 25%, 14.9%, and 4.7% of the expression level in females, respectively (Fig. 4). The results of *in situ* hybridization showed that *Ab-far*-1 mRNA was present in the hypodermis of juveniles (Fig. 5C), the hypodermis and ovaries of females (Fig. 5 D, E), and in the hypodermis and testes of males (Fig. 5F). No hybridization signal was detected in the nematodes after incubation with the control sense probe (Fig. 5A, B).

### The effect of RNAi

**Figure 6:**
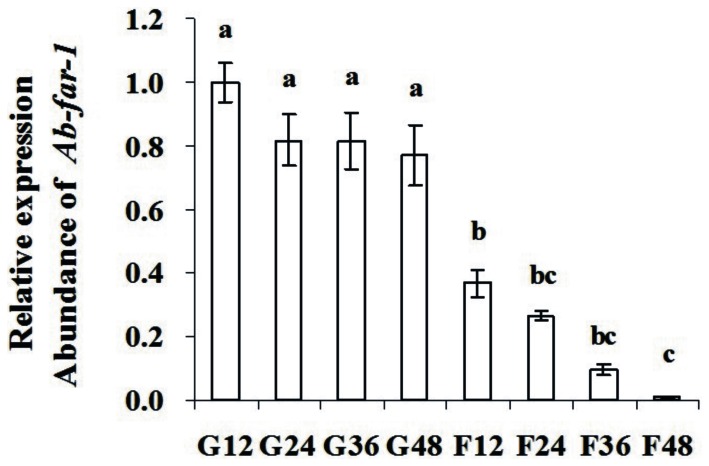
Expression of the *Ab-far-1* mRNA in *Aphelenchoides besseyi* treated with *Ab-far-1* double-stranded (ds) RNA. G12, G24, G36 and G48 indicates expression of *Ab-far-1* mRNA in control nematodes soaked by non-endogenous *gfp* dsRNA solution for 12 h, 24 h, 36 h and 48 h, respectively. F12, F24, F36 and F48 indicates expression of *Ab-far-1* mRNA in nematodes soaked by *Ab-far-1* dsRNA for 12 h, 24 h, 36 h and 48 h, respectively. Bars indicate standard errors of mean data (n = 3) and different letters indicate significant differences (*p*<0.01) between treatments.

After treatment with *Ab-far-1* dsRNA, the expression of *Ab-far-1* mRNA in *A. besseyi* was detected by the qPCR. Compared with the relative expression level of *Ab-far-1* mRNA in the corresponding control nematodes (*gfp* dsRNA treatment), the relative expression of *Ab-far-1* mRNA in nematodes treated with *Ab-far-1* dsRNA was reduced by 63.2% at 12 h, 55.1% at 24 h, 71.8% at 36 h, and 76% at 48 h (Fig. 6). The expression level of *Ab-far-1* mRNA was reduced with the increasing incubation time with dsRNA, while the non-endogenous *gfp* dsRNA control had no effect on the expression of *Ab-far-1* mRNA at different time points.

**Figure 7:**
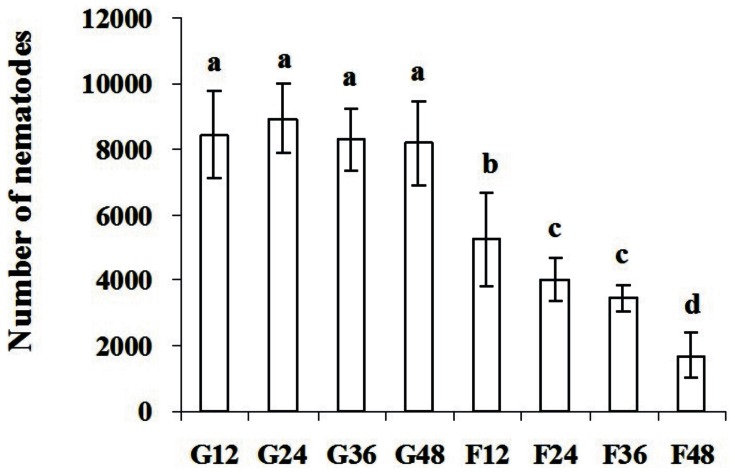
Number of *Aphelenchoides besseyi* on carrot callus after inoculation of 30 females 35 days later, respectively. G12, G24, G36 and G48 indicates number of *A. besseyi* after inoculating 30 females treated by non-endogenous *gfp* dsRNA solution for 12 h, 24 h, 36 h and 48 h, respectively. F12, F24, F36 and F48 indicates number of *A. besseyi* after inoculating 30 females treated by *Ab-far-1* dsRNA for 12 h, 24 h, 36 h and 48 h, respectively. Bars indicate standard errors of mean data (n = 5) and different letters indicate significant differences (*p*<0.01) among treatments.

After being treated with dsRNA for 12 h, 24 h, 36 h and 48 h and inoculated onto carrot callus for 35 days, *A. besseyi* treated with *Ab-far-1* dsRNA had significantly (*p*<0.01) lower reproduction than those treated with *gfp* dsRNA at all time points. The reproduction of nematodes was significantly (*p*<0.01) decreased with the increasing exposure time to *Ab-far-1* dsRNA. The reproduction of nematodes treated with *gfp* dsRNA did not show difference at different treatment time points (Fig. 7).

## Discussion

FAR protein is a specific protein only in the nematodes, which plays a critical role in the development and reproduction in nematodes, and in the processes of infecting host [7], [15], [27]. In this study, we obtained the full-length sequences of *Ab-far-1* in *A. besseyi*, which is the first report for the FAR gene in Aphelenchida nematodes. The secondary structure of *Ab*-FAR-1 was rich in α-helix but without β extended structure, which is an important factor that sets FAR apart from the similar lipid-binding proteins of vertebrates [17], [19], [28], [29], [30], [31]. There are four conserved Proline residues in *Ab*-FAR-1 and other six FAR proteins amino acid sequences in sequence alignment. Proline is an inclined interrupt helical structure and cannot generate the hydrogen bond to sustain the conformation of α-helix and is an amino acid which requires less free energy to fold [32]. But there is Glutamic or Alanine residue behind the two Proline residues in the middle of first helix. The Glutamic and Alanine are the strongest nucleate α-helices [33], which may influence the secondary structure of FAR. It has been reported that FAR proteins are secreted from the cell to the extracellular environment where they presumably play roles in the sequestration and transport of fatty acids and retinoids to maintain the nematodes normal life activity during the infection to the host [9], [13], [15], [17]. A hydrophobic secretory signal peptide was identified in the *Ab*-FAR-1 sequences, which indicates *Ab*-FAR-1 may be secreted from the cell to the extracellular environment. Furthermore, the *Ab*-FAR-1 also contained a conserved casein kinase II phosphorylation site, which is appeared in all known nematodes FAR proteins [13], [15], [17], [19]. Phosphorylation is known to regulate the biological activities of many proteins, including gene regulation, homodimerisation control and the stability of α-helices [34], [35], [36], [37], [38], [39]. Thus we assume that the phosphorylation sites may play an important role in the process of the infection of *A. besseyi* to the hosts.

It is believed that FAR protein is necessary for the nematodes not only to bind lipids like linoleic and linolenic acids to maintain the metabolism, destroy the plant defense systems and complete the infection, but also to bind retinoid in collagen synthesis, embryonic development and reproduction [8], [15]. In this study, the expression of *Ab-far-1* mRNA at different nematode stages showed the expression was highest in females and lowest in males, higher in eggs than in juveniles, which conformed to their individual biological functions. The female of *A. besseyi* plays key roles in infection and reproduction, while the number and infection ability of male is lower than female and the male is not necessary for the reproduction of the nematode as it is capable of parthenogenesis. The egg and juvenile are responsible for reproduction and infection, respectively, which indicates that this protein may play an important role in the formation of the embryo and cell differentiation. It was also found that the FAR gene expression at different developmental stages was associated the biological functions in animal parasitic nematodes. The *Hc-FAR-1* gene expression of *Haemonchus contortus* was higher in adult than in larvae [27] and *AceFAR-1* mRNA expression was lowest in male than other developmental stages of *A. ceylanicu* [5]. In plant parasitic nematodes *G. pallida*, Prior [15] reported the *gp*-*far*-1 mRNA was present in the second-stage juveniles, virgin females and adult female, but did not analyze the differences of its expression level.

*In situ* hybridization results revealed that *Ab-far-1* mRNA was present in the hypodermis of *A. besseyi*. The cuticle layer, hypodermis and internal muscle layer form the nematode body wall, and the hypodermis is one of the most active areas in the nematodes. The expression of *Ab-far-1* in the hypodermis may help nematodes to utilize the fatty acid and retinoid from the hosts and the environment quickly to maintain the autologous metabolism. Meanwhile, *Ab-far-1* in the hypodermis may help nematodes to neutralize plant defense and to complete the infection. The *gp*-*far*-1 mRNA was localized in the hypodermis of the infectious second-stage juveniles of *G*. *pallida* [15]. In addition, *Ab-far-1* mRNA was also present in the ovaries of females and the testes of males, but signals appeared diffuse, whether is related to the internal structure of ovaries and testes is not clear because the nematodes were cut off, fixed and hybridized *in situ* hybridization, it needs further study to be confirmed.

In this paper, RNAi was employed to further verify the *Ab-far-1* function. To our knowledge, it has not been reported in other nematodes FAR gene study. After the treatment with *Ab-far-1* dsRNA, the expression of *Ab-far-1* and the reproduction of nematodes were decreased with the increasing exposure time. After treatment with dsRNA for 48 h, the silencing efficiency of *Ab-far-1* was the maximum and the reproduction of nematodes was the least. This may be because the FAR protein can bind with fatty acids and retinol. Since the retinol is thought to be essential for collagen synthesis and embryonic development, down-regulation of *Ab-far-1* by RNAi leads to less binding with retinol, and thus causes the reduced reproduction in *A. besseyi*. This evidence further proves the roles of *Ab*-FAR-1 in the development and reproduction of *A. besseyi*.

The transgenic plant with the expression of specific dsRNA to the target gene in nematodes would result in the inhibition of target gene in nematodes after feeding [40]. Thus, the expression of specific dsRNAs for nematode development-related and parasitic genes in transgenic plants is a potentially way to enhance the resistance to nematodes and to control plant parasitic nematodes. FAR protein is not only a kind of protein related to development, reproduction, infection and interfered the plant defense system, but also a specific protein only existed in the nematodes [7], [27]. Moreover, the *Ab*-FAR-1 gene has not been found in mammal and other animals, so this transgenic plant containing *Ab*-FAR-1 dsRNA will not cause any safety issue to the environment and other organisms. Therefore, it is imperative to consider the generation of transgenic plant containing *Ab*-FAR-1 dsRNA, which will be an effective method to control *A. besseyi*.

## Supporting Information

Figure S1 **EST sequence of** ***Aphelenchoides besseyi*** **fatty acid and retinoid binding protein gene.**
(TIF)

Figure S2 ***Ab-far-1*** **cDNA sequence and its deduced amino acid sequence.** *atg, Translation starting signal. taa, Translation termination signal. Nucleotide sequence are denoted in lowercase and deduced amino acid sequence are marked in dark gray.*
(TIF)

Figure S3 **The structural predictions of** ***Aphelenchoides besseyi Ab*** **-FAR-1 and its amino acid sequence alignments with other nematode FAR proteins.** *Ov*-FAR-1, *Onchocerca volvulus* FAR (Q25619); *Bm*-FAR-1, *Brugia malayi* FAR (Q93142); *Gp*-FAR-1, *Globodera pallida* FAR (CAA70477); *Ce*-FAR-1, *Caenorhabditis elegans* FAR-1 (NP 499010); *Ce*-FAR-2, *C. elegans* FAR-2 (NP 499011); *Ce*-FAR-6, *C. elegans* FAR-6 (NP 502561); *Ab*-FAR-1, *A. besseyi* FAR-1; Lowercase, putative secretory signal peptides; shaded boxes, positions where amino acids are conserved in all the sequences; underscored, consensus N-linked glycosylation sites; boldface, conserved casein kinase II phosphorylation site; In Consensus line, uppercase amino acids which are conserved at that position in all of the sequences, lowercase amino acid which are conserved at that position in more than half of the sequences, # indicates any of NDQEBZ. The Jpred line shows the secondary strucure prediction from submission of the multiple alignment to the Jpred secondary structure prediction programme. H Prediction for α-helix; gaps regions for which no structural prediction emerged. No β-structure was predicted by Jpred or any other secondary structure prediction programmes.
(TIF)

Figure S4 **Sodium dodecyl sulphate polyacrylamide gel of purified His-** ***Ab*** **-FAR-1 and r** ***Ab*** **-FAR-1.** Lane 1, purified His-*Ab*-FAR-1; Lane 2, purified r*Ab*-FAR-1; Lane M, PageRuler Prestained protein Ladder (Sofar Technology, China).
(TIF)
