# Supplementary figures and images for: Molecular Characterization and Functions of Fatty Acid and Retinoid Binding Protein Gene (Ab-far-1) in Aphelenchoides besseyi
Source: PLoS One. 2013 Jun 5;8(6):e66011. doi: 10.1371/journal.pone.0066011 (PMC3673936; doi:10.1371/journal.pone.0066011)

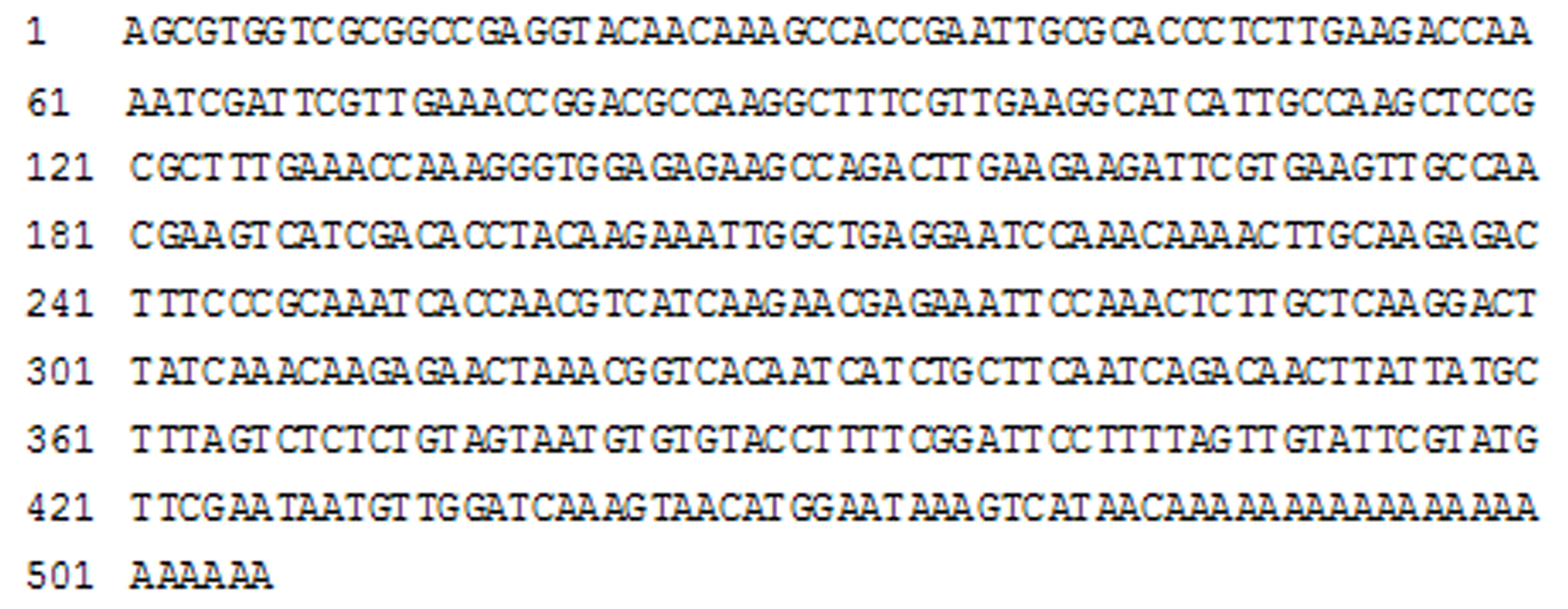

Supplement: Figure S1 — EST sequence of Aphelenchoides besseyi fatty acid and retinoid binding protein gene. (TIF) [file pone.0066011.s001.tif]

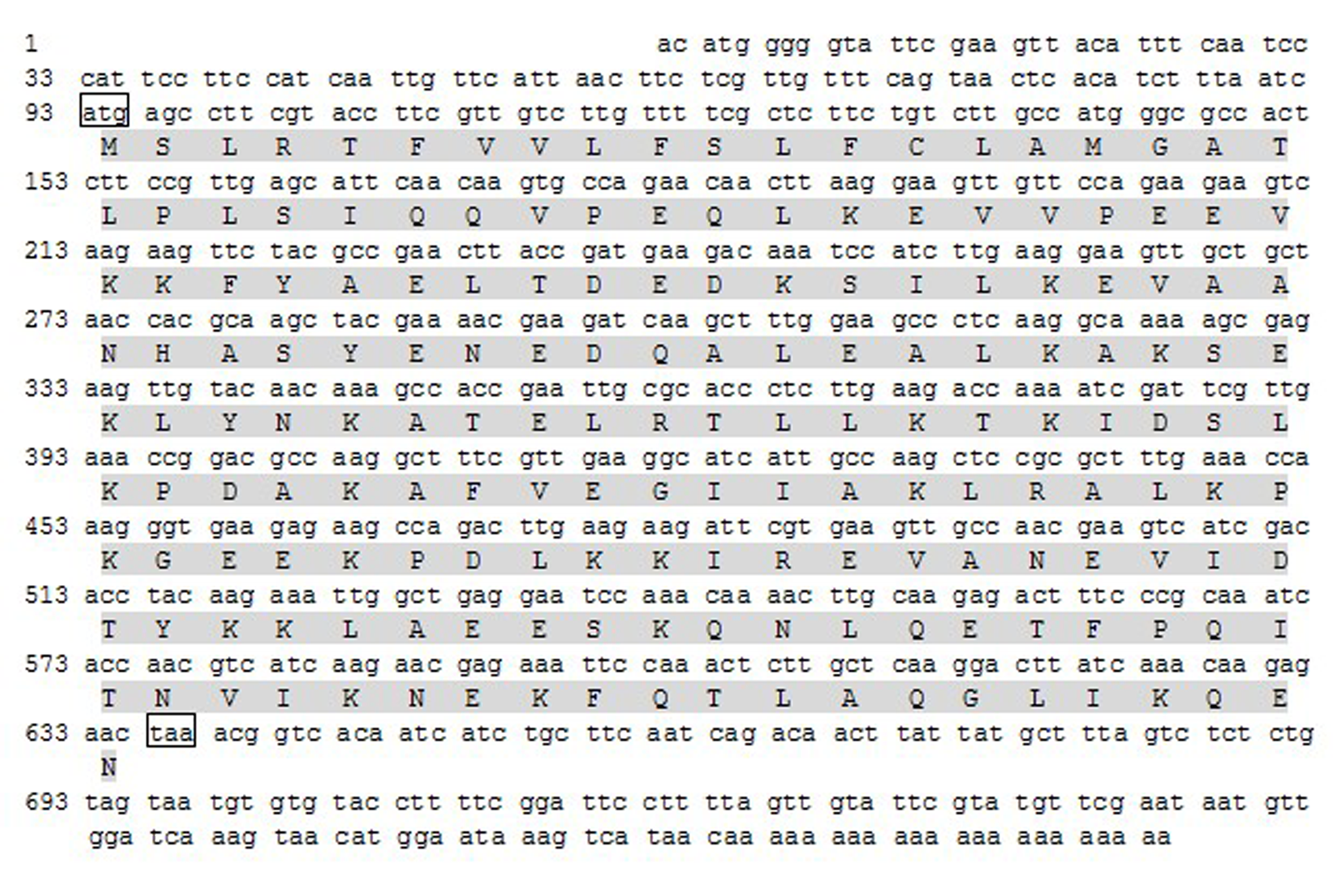

Supplement: Figure S2 — Ab-far-1 cDNA sequence and its deduced amino acid sequence. atg, Translation starting signal. taa, Translation termination signal. Nucleotide sequence are denoted in lowercase and deduced amino acid sequence are marked in dark gray. (TIF) [file pone.0066011.s002.tif]

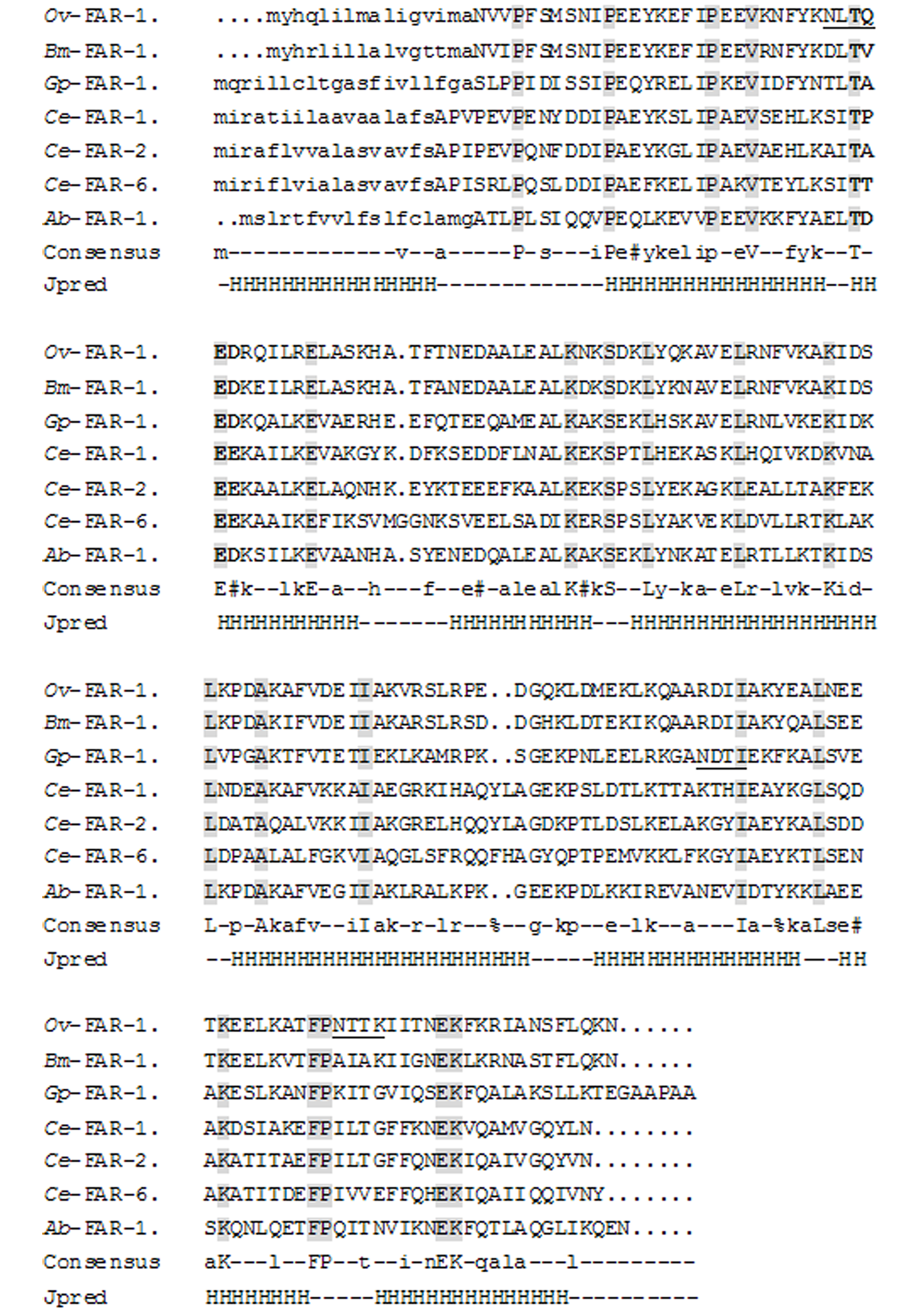

Supplement: Figure S3 — The structural predictions of Aphelenchoides besseyi Ab -FAR-1 and its amino acid sequence alignments with other nematode FAR proteins. Ov-FAR-1, Onchocerca volvulus FAR (Q25619); Bm-FAR-1, Brugia malayi FAR (Q93142); Gp-FAR-1, Globodera pallida FAR (CAA70477); Ce-FAR-1, Caenorhabditis elegans FAR-1 (NP 499010); Ce-FAR-2, C. elegans FAR-2 (NP 499011); Ce-FAR-6, C. elegans FAR-6 (NP 502561); Ab-FAR-1, A. besseyi FAR-1; Lowercase, putative secretory signal peptides; shaded boxes, positions where amino acids are conserved in all the sequences; underscored, consensus N-linked glycosylation sites; boldface, conserved casein kinase II phosphorylation site; In Consensus line, uppercase amino acids which are conserved at that position in all of the sequences, lowercase amino acid which are conserved at that position in more than half of the sequences, # indicates any of NDQEBZ. The Jpred line shows the secondary strucure prediction from submission of the multiple alignment to the Jpred secondary structure prediction programme. H Prediction for α-helix; gaps regions for which no structural prediction emerged. No β-structure was predicted by Jpred or any other secondary structure prediction programmes. (TIF) [file pone.0066011.s003.tif]

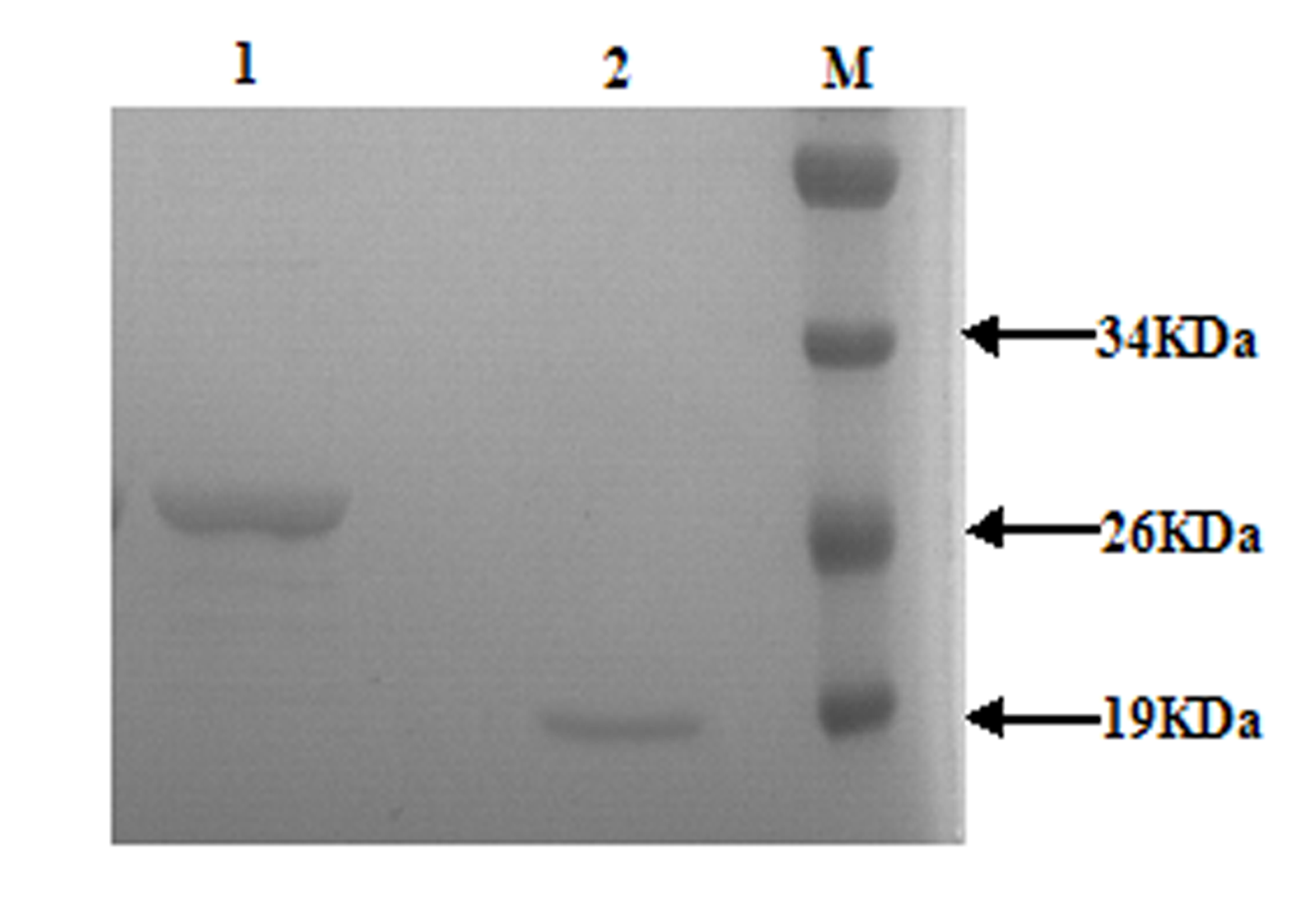

Supplement: Figure S4 — Sodium dodecyl sulphate polyacrylamide gel of purified His- Ab -FAR-1 and r Ab -FAR-1. Lane 1, purified His-Ab-FAR-1; Lane 2, purified rAb-FAR-1; Lane M, PageRuler Prestained protein Ladder (Sofar Technology, China). (TIF) [file pone.0066011.s004.tif]
